# Supplementary material for: Spatial non-parametric Bayesian clustered coefficients
Source: Sci Rep. 2024 Apr 27;14:9677. doi: 10.1038/s41598-024-59973-w (PMC11055928; doi:10.1038/s41598-024-59973-w)
Supplement: Supplementary file 1 — Supplementary Information. [file 41598_2024_59973_MOESM1_ESM.pdf]

S1. Appendix. Blocked MCMC for a Spatial Dirichlet Process Mixture Model (SDPMM)

In this section, we use a blocked sampler. This method updates groups of parameters all at once to make computations more efficient. By updating these parameter blocks together, we can achieve faster convergence and enhance the overall performance of the chain.
Given the model specifications, the joint distribution (prior to normalization) can be expressed as:

$$p(y(s), Z, \beta(s), \mu, \Sigma, \sigma^2(s), V, b) = p(y(s)|\beta(s), Z, \sigma^2(s)) \times p(Z|V) \times p(\beta(s)|\mu, \Sigma, Z) \times p(\mu|\Sigma) \times p(\Sigma) \times p(\sigma^2(s)) \times p(V) \times p(b)$$

Block Gibbs Sampling

- **Initialization**

  Choose initial values for $\beta(s), \mu, \Sigma, \sigma^2(s), V, b$ and latent variables Z.

- **Gibbs Sampling Iterations**

  A. Sample Z given other parameters and data:

  For each observation i, sample Z_i from its categorical distribution based on likelihood and prior terms:

  $$p(Z_i = k|y(s), \beta(s), \mu, \Sigma, \sigma^2, V, b) \propto p(y(s)|\beta(s_{z_i}), x(s), \sigma^2(s)) \times p(Z_i = k|V)$$

  B. Sample other blocks of parameters:

  Sample $\beta(s)$ given $Z, \mu, \Sigma, y(s), x(s)$ and other relevant variables:

  $$p(\beta(s)|y(s), Z, \mu, \Sigma, \sigma^2(s), V, b) \propto p(y(s)|Z, \beta(s), \sigma^2(s)) \times p(\beta(s)|\mu_Z, \Sigma_Z)$$

  C. Compute the gradient of the log posterior with respect to $\beta(s)$ using the derived gradients from the likelihood and prior terms:

  $$\frac{\partial}{\partial \beta(s)} \log p(\beta(s), Z, \mu, \Sigma, \sigma^2(s), V, b|y(s)) = \sum_i 2x(s)(y(s) - x^T(s)\beta(s_{z_i}))$$

  D. Update $\beta(s)$ using the gradient information and suitable sampling algorithm (e.g., Metropolis-Hastings).

  E. Sampling μ and Σ:

  Given the conjugacy in the model, μ_k and Σ_k can be jointly sampled from their respective full conditional distributions.

  F. For each cluster k, compute the posterior distribution of μ_k and Σ_k using the corresponding data and prior:

  $$p(\mu_k, \Sigma_k|\text{data and prior information}) \propto p(\beta(s_{z_i})|\mu_k, \Sigma_k) \times p(\mu_k|\Sigma_k) \times p(\Sigma_k)$$

  G. Update μ and Σ using the sampled values from their posterior distributions.

  H. Sample $\sigma^2(s)$ given $y(s), x, \beta(s), Z$, and other variables:

  For each spatial location s, sample $\sigma^2(s)$ using the inverse-gamma distribution:

  $$\sigma^2(s) \sim \text{Inverse-Gamma}(\alpha_1, \alpha_2)$$

  I. Sample V and b given their priors and their relationship to Z:

  V_k represents the parameter associated with latent variable assignment for cluster k. It determines the probabilities of assigning observations to cluster k. This step updates the assignment probabilities based on the data and relationships between V and the latent variables Z.

  Sample V_k using the beta distribution with updated parameters based on the data and relationship to Z:

  $$V_k \sim \text{Beta}(a_v, b_v)$$
